# The complete chloroplast genome and phylogentic results support the species position of *Swertia banzragczii* and *Swertia marginata* (Gentianaceae) in Mongolia

**DOI:** 10.1186/s40529-024-00417-z

**Published:** 2024-04-24

**Authors:** Dashzeveg Oyuntsetseg, Nudkhuu Nyamgerel, Shukherdorj Baasanmunkh, Batlai Oyuntsetseg, Magsar Urgamal, Jung Won Yoon, Gun-Aajav Bayarmaa, Hyeok Jae Choi

**Affiliations:** 1 Department of Biology, School of Arts and Sciences, National University of Mongolia, 14201 Ulaanbaatar, Mongolia; 2 Department of Biology and Chemistry, Changwon National University, 51140 Changwon, South Korea; 3 Laboratory of Plant Taxonomy and Phylogenetic, Botanic Garden and Research Institute, Mongolian Academy of Sciences, 13330 Ulaanbaatar, Mongolia; 4 DMZ Botanic Garden, Korea National Arboretum, 11186 Pocheon, South Korea

**Keywords:** *Swertia*, Endemic, Chloroplast genome, Taxonomy, Flora of Mongolia

## Abstract

**Background:**

*Swertia banzragczii* and *S. marginata* are important medicinal species in Mongolia. However, their taxonomic positions and genetic backgrounds remain unknown. In this study, we explored the complete chloroplast genomes and DNA barcoding of these species and compared them with those of closely related species within the subgenus to determine their taxonomic positions and phylogenetic relationships.

**Result:**

The chloroplast genomes of *S. banzragczii* and *S. marginata* encoded 114 genes, including 80 protein-coding genes, 30 tRNA genes, and 4 rRNA genes. Among them, 16 genes contained a single intron, and 2 genes had two introns. Closely related species had a conserved genome structure and gene content. Only differences in genome length were noticed, which were caused by the expansion and contraction of the inverted repeat (IR) region and loss of exons in some genes. The *trn*H-GUG–*psb*A and *trn*D-GUC–*trn*Y-GUA intergenic regions had high genetic diversity within *Swertia* plastomes. Overall, *S. banzragczii* and *S. marginata* are true species and belong to the subgenus *Swertia*.

**Conclusions:**

These results provide valuable genetic and morphological information on rare and subendemic *Swertia* species in Mongolia, which can be used for further advanced studies on the *Swertia* genus.

## Background

*Swertia* L. is one of the largest genera in the Gentianaceae Juss. and comprises 160 species (Ho and Liu 2015; Nampy et al. 2015; POWO 2023). Members of the *Swertia* genus are mainly distributed in the temperate and alpine regions of Asia, Europe, and the Americas (Ho and Liu 2015). This genus has great medicinal importance and is the most taxonomically challenging taxon in the family, largely because of the morphological similarities of species within this genus and closely related genera (Chassot et al. 2001; Ho and Liu 2015; Cao et al. 2022). It is distinguished by a rotated corolla and the presence of coralline nectariferous glands in related genera within the family (Ho and Liu 2015). Furthermore, the nature and shape of these glands are two of the main diagnostic characteristics for species delimitation in this genus (Favre et al. 2015).

Four to five species of *Swertia* have been recorded in Mongolia, i.e., *Swertia banzragczii* Sanchir, *S. marginata* Schrenk (or *S. komarovii* Pissajuk.), *S. obtusa* Ledeb. and *S. dichotoma* L. (Baasanmunkh et al. 2022a). The current taxonomic status of *S. banzragczii* and *S. marginata* (or *S. komarovii* Pissajuk.) is still doubtful (Ho and Liu 2015). However, these species are currently accepted taxa on websites such as the Global Biodiversity Information Facility (GBIF) and Plants of the World Online (POWO 2023). The two *Swertia* species have not been fully investigated based on especially molecular evidence, including DNA barcoding and plastomes.

The development of barcoding markers has greatly advanced our understanding of the relationships between morphologically similar species. Nuclear ribosomal DNA internal transcribed spacer (ITS) and *mat*K, *trn*L, *trn*L-F, and *trn*S of chloroplast markers mainly used in the phylogenetic study of *Swertia*; however, it has been revealed that *Swertia* is strongly paraphyletic to other genera within the Gentianaceae family (Chassot et al. 2001; Xi et al. 2014). Therefore, more genomic studies are needed for reconstructing the phylogenetic relationships of *Swertia*. Recently, the chloroplast genome has been widely used in plant system evolution, related species identification, genetic diversity analysis, chloroplast genetic engineering, and other applications due to the development of high-throughput sequencing technology (Yang et al. 2016; Li et al. 2021; Fang et al. 2023; Nyamgerel et al. 2023, 2024). The chloroplast genome (plastome) has a conserved structure, encodes 120–130 genes, most of which encode part of the photosynthetic apparatus of the organelle (Jensen and Leister 2014), and is maternally inherited (Wicke et al. 2011). Therefore, plastomes have stronger species-discriminating abilities and provide more information on genetic variation than ultra-barcodes (Hollingsworth et al. 2016). Several studies have explored the complete chloroplast genome of *Swertia* species, mainly from China (Bi et al. 2020; Du et al. 2022; Yang et al. 2020, 2022, 2023; Cao et al. 2022). These studies determined the plastome structure, genetic variety, and phylogenetic relationships across 26 species of *Swertia* and compared them with those of the subtribe Swertiinae species. In addition, the ITS marker is widely used for phylogenetic studies and ITS region of many *Swertia* species was sequenced. The ITS is biparentally inherited and has a higher mutation rate than maternally inherited plastome (Wolfe et al. 1987). However, little is known about the comparative plastome analysis with phylogenetic relationships of *Swerita* species in Mongolia. To address this gap, therefore, we investigated the plastome analysis and DNA barcoding on previously poorly known two Mongolian *Swertia* species.

In this study, we pursued the following aims: (i) to explore the complete chloroplast genomes of *S. banzragczii* and *S. marginata*; (ii) to identify the candidate regions from chloroplast genome to use as barcoding markers; (iii) compared chloroplast genome and barcoding region to determine the taxonomic positions and phylogenetic relationships of the closely related species.

## Results

### The chloroplast genomes of *S. banzragczii* and *S. marginata*

**Fig. 1:**
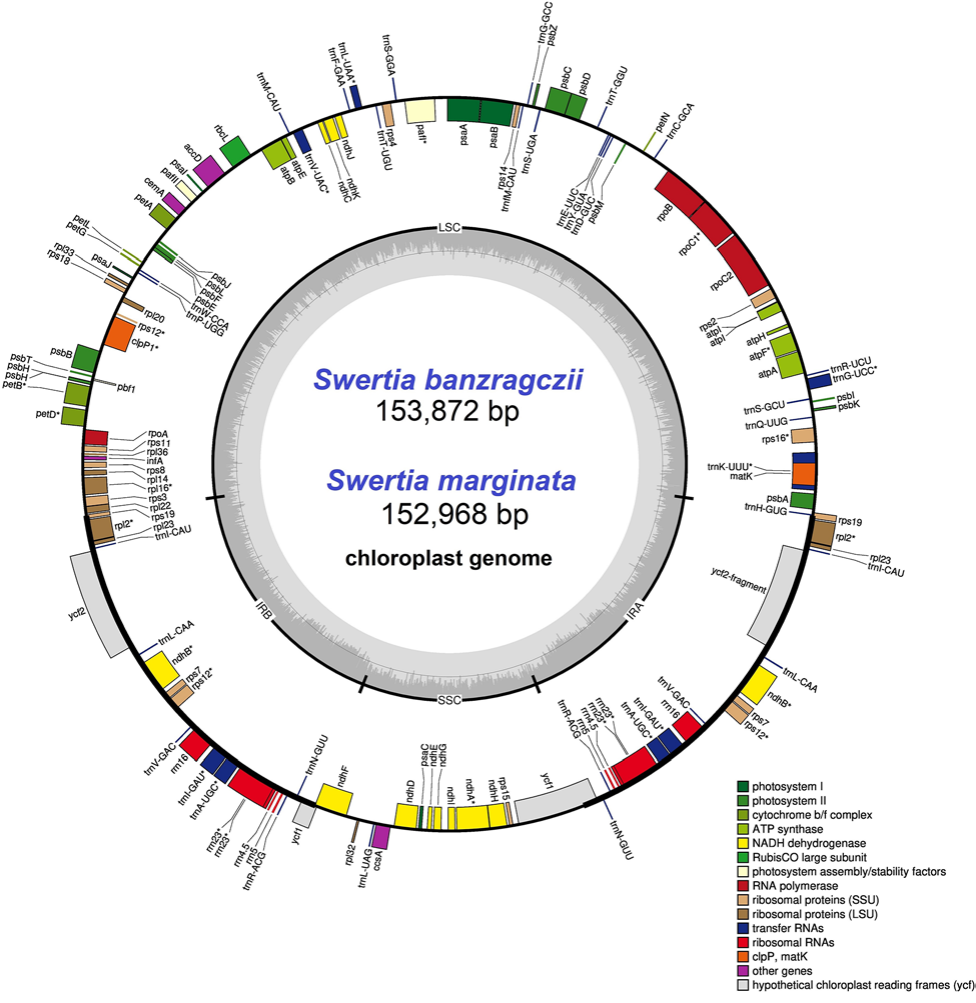
Circular map of the *S. banzragczii* and *S. marginata* complete chloroplast genomes. The inner ring is divided into four areas: SSC, IRb, LSC, and IRa. The inner ring indicates the GC content (dark gray or green) or the AT content (light gray or green). The genes in the outer ring region are transcribed clockwise, while those in the inner ring are transcribed counterclockwise. The legend classifies the cp. genes based on their functions

The chloroplast genomes of *S. banzragczii* and *S. marginata* were sequenced for the first time. In total, 54,848,640 and 66,783,272 reads were produced for *S. banzragczii* and *S. marginata*, respectively (Table 1). After *de novo* assembly, a single contig was generated for each plastome. The two plastomes had typical quadripartite natural structures comprising a large single copy (LSC), a pair of inverted repeats (IR), and a small single copy (SSC) (Fig. 1). The total length of *S. marginata* plastome was shorter than the *S. banzragczii* plastome in all regions (Table 1). The overall guanine-cytosine (GC) content of the two plastomes was similar, differing by 0.1% in total length. The two plastomes encoded 114 genes, including 80 protein-coding genes, 30 tRNA genes, and 4 rRNA genes. Among them, 18 genes (7 protein-coding, 7 tRNA, and 4 rRNA genes) were duplicated in the IR regions. Sixteen genes contained a single intron, and two genes had two introns (Table 2). Of the 18 genes, 12 intron-containing genes (*trn*K-UUU, *rps*16, *trn*G-UCC, *atp*F, *rpo*C1, *trn*L-UAA, *trn*V-UAC, *pet*B, *pet*D, and *rpl*16) were located in the LSC and 1 gene (*ndh*A) was located in the SSC. Five genes (*rpl*2, *ndh*B, *rps*12, *trn*I-GAU, and *trn*A-UGC) were duplicated in the IRs. The *rps*12 gene was a trans-spliced gene with 5′ end located in the LSC and 3′ end located in the IR.

**Table 1:** Raw reads and genome assembly information for *S. banzragczii* and *S. marginata* plastomes

	*S. banzragczii*	*S. marginata*
Input reads (bp)	54,848,640	66,783,272
Trimmed reads (bp)	7,153,975,259	7,538,583,714
Total raw base (bp)	8,282,144,640	10,084,274,072
Trimmed bases (bp)	47,559,948	50,265,678
Q20 (%)	99.23	98.95
Q30 (%)	93.54	95.89
Coverage (%)	100	100
Depth (X)	46.493	49,282
CG content (%)	38.0	38.1
Cp genome length (bp)	153,872	152,968
LSC/GC content	83,841/36.1	83,216/36.1
IR/GC content	25,855/43.3	25,755/43.4
SSC/GC content	18,321/31.8	18,242/31.9

**Table 2:** Gene category of *S. banzragczii* and *S. marginata* plastomes

Category	Group of genes	Name of genes
RNA genes	ribosomal RNA genes (rRNA)	*rrn*5^c^, *rrn*4.5^c^, *rrn*16^c^, *rrn*23^a, c^
	Transfer RNA genes (tRNA)	*trn*A-UGC^a, c^, *trn*C-GCA, *trn*D-GUC, *trn*E-UUC, *trn*F-GAA, *trn*fM-CAU, *trn*G-GCC, *trn*G-UCC^a^, *trn*H-GUG, *trn*I-CAU^c^, *trn*I-GAU^a^, *trn*K-UUU^a^, *trn*L-CAA^c^, *trn*L-UAA^a^, *trn*L-UAG, *trn*M-CAU, *trn*N-GUU^c^, *trn*P-UGG, *trn*Q-UUG, *trn*R-ACG^c^, *trn*R-UCU, *trn*S-GCU, *trn*S-GGA, *trn*S-UGA, *trn*T-GGU, *trn*T-UGU, *trn*V-GAC^c^, *trn*V-UAC^a^, *trn*W-CCA, *trn*Y-GUA
Ribosomal proteins	Small subunit of ribosome	*rps*2, *rps*3, *rps*4, *rps*7^c^, *rps*8, *rps*11, *rps*12^c, b, d^, *rps*14, *rps*15, *rps*16^a^, *rps*18 *rps*19^c^
Transcription	Large subunit of ribosome	*rpl*2^a, c^, *rpl*16^a^, *rpl*14, *rpl*20, *rpl*22, *rpl*23^c^, *rpl*33, *rpl*36, *rpl*32
	RNA polymerase	*rpo*A, *rpo*B, *rpo*C1^a^, *rpo*C2
Protein genes	Photosystem I	*psa*A, *psa*B, *psa*C, *psa*I, *psa*J, *ycf*3^b^, *ycf*4
	Photosystem II	*psb*A, *psb*B, *psb*C, *psb*D, *psb*E, *psb*F, *psb*H, *psb*I, *psb*J, *psb*K, *psb*L, *psb*M, *psb*N, *psb*T, *psb*Z
	Cytochrome b6/f	*pet*A, *pet*B^a^, *pet*D^a, d^, *pet*G, *pet*L, *pet*N
	ATP synthase	*atp*A, *atp*B, *atp*E, *atp*F^a^, *atp*H, *atp*I
	Rubisco	*rbc*L
	NADH dehydrogenase	*ndh*A^a^, *ndh*B^a, c^, *ndh*C, *ndh*D, *ndh*E, *ndh*F, *ndh*G, *ndh*H, *ndh*I, *ndh*J, *ndh*K
	ATP-dependent protease subunit P	*clp*P^b^
	Chloroplast envelope membrane protein	*cem*A
	Transitional initiation factor	*inf*A
Other genes	Maturase	*mat*K
	Subunit acetyl-coA carboxylase	*acc*D
	C-type cytochrome synthesis	*ccs*A
	Hypothetical proteins	*ycf*1^c^, *ycf*2^c^, *ycf*15^e, c^
	Component of TIC complex	*ycf*3^b^

^a^Gene with one intron, ^b^Gene with two introns, ^c^Gene with copies, ^d^Trans-splicing gene

### Chloroplast genome comparison of genus *Swertia* species

**Fig. 2:**
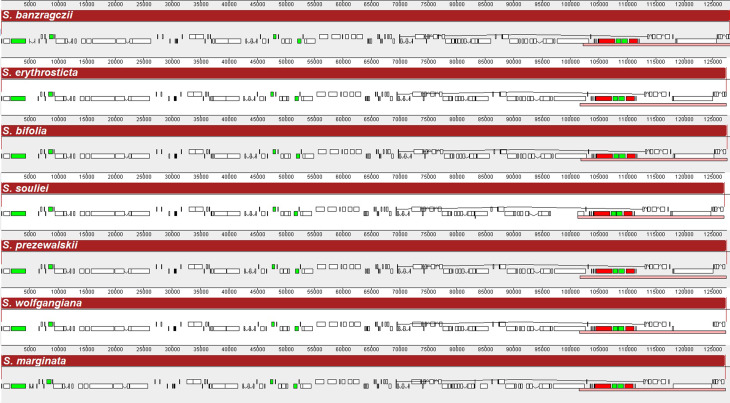
Comparison of complete plastomes from seven *Swertia* species using the MAUVE alignment. Blocks in the top of each row are in the same orientation, while blocks on the bottom of each row are in the inverse orientation. Boxes under each genome map represent protein-coding genes (white), rRNAs (red), and tRNAs (green). Red blocks in the lowest bottom row are inverted repeat (IR) regions

**Fig. 3:**
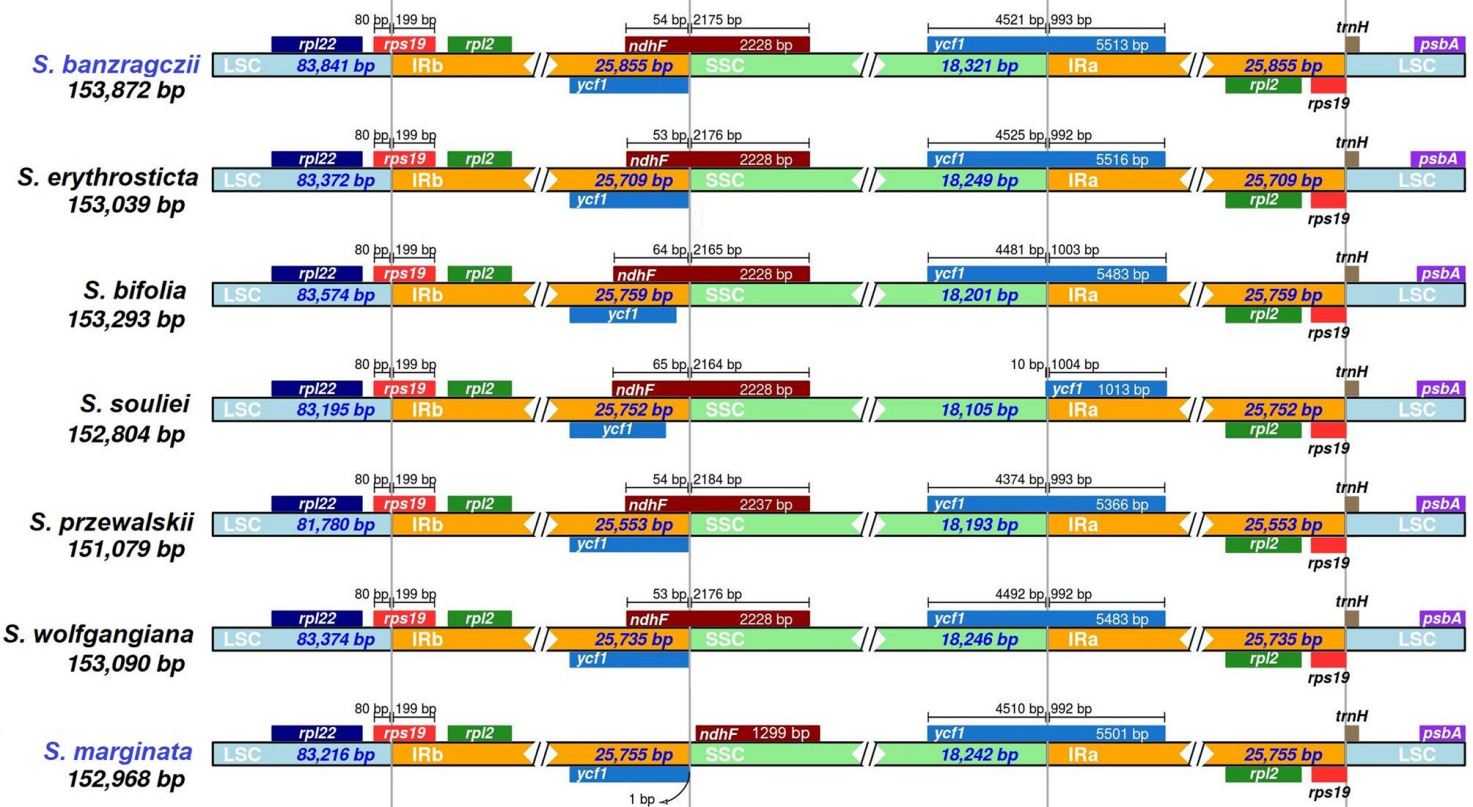
Comparison of LSC, IR, and SSC junction positions of *S. banzragczii* and *S. marginata* plastomes. LSC, large single-copy region; SSC, small single-copy region; IR, inverted repeat

**Fig. 4:**
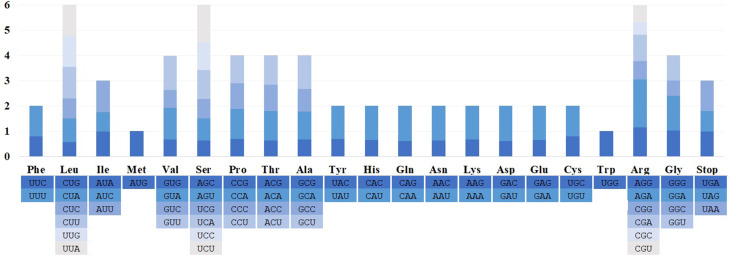
Codon usage for 20 amino acids in the protein-coding genes of *S. banzragczii* and *S. marginata*. The colors in the bars are the same as that of the codons below the figure. RSCU: relative synonymous codon usage

**Fig. 5:**
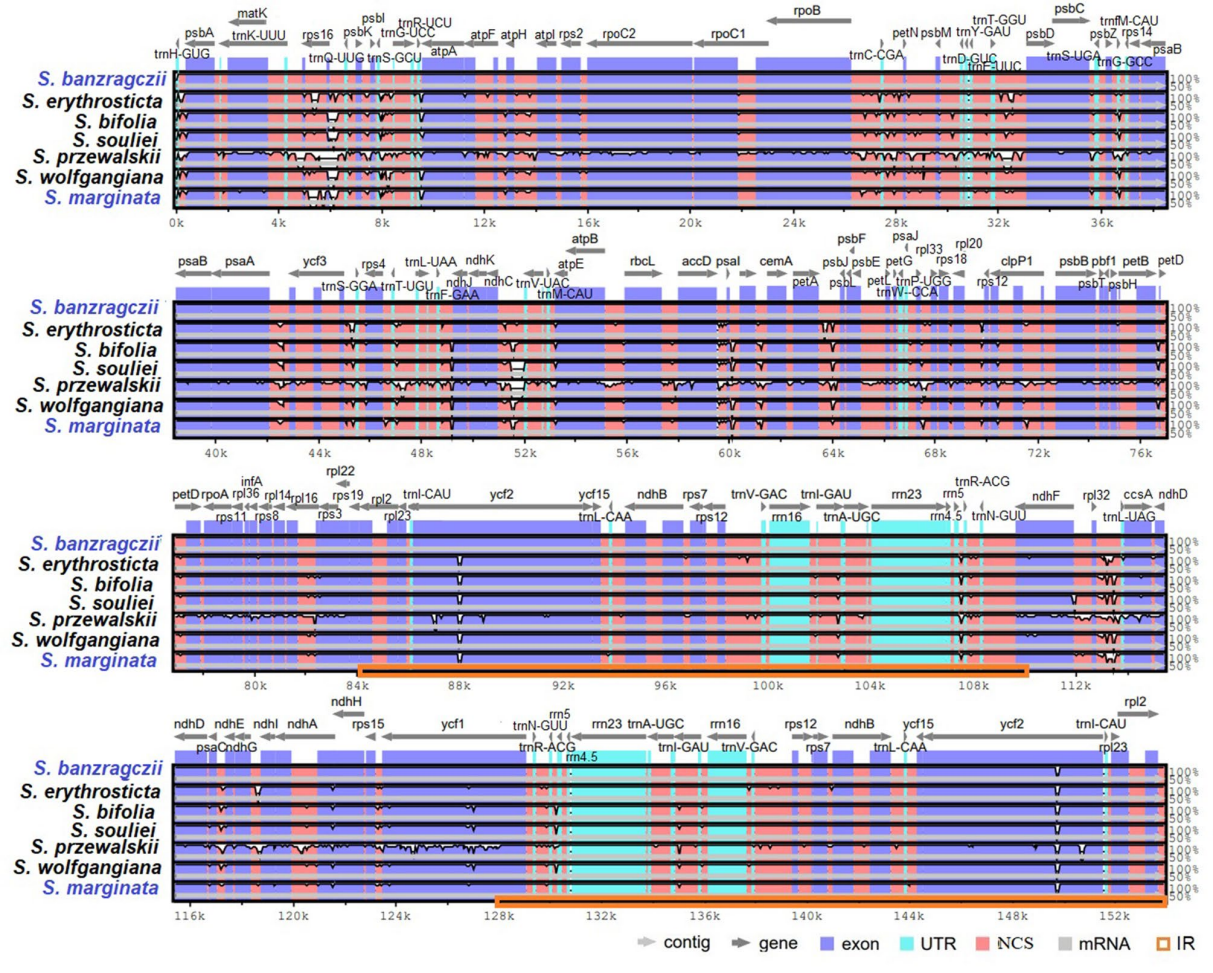
Comparison of complete plastomes from seven *Swertia* species, including *S. banzragczii* as a reference, using the mVISTA software. The gray arrows above the alignment indicate genes. Different colors represent different regions (coding and non-coding). The horizontal axis indicates the coordinates within the plastomes. The vertical scale represents the percentage of identity, ranging from 50 to 100%

The cp. genome size, structure, and gene positions of *S. banzragczii* and *S. marginata* were compared with those of five closely related species within the subgenus *Swertia*: *S. bifolia* Batalin, *S. erythrosticta* Maxim., *S. souliei* Burkill, *S. przewalskii* Pissjauk, and *S. wolfgangiana* Grüning. The gene content and location in all regions of closely related species were similar, and the plastomes did not display any species-specific inversion or translocation (Fig. 2). The IR of *S. banzragczii* and *S. marginata* were 25,855 and 25,755 bp, respectively. The borders between the IR regions and two single-copy regions (LSC and SSC) were similar to closely related species (Fig. 3). The *rps*19, *ndh*F, and *ycf*1 genes spanned the LSC/IRb, IRb/SSC, and SSC/IRa junctions, respectively. However, the *ndh*F gene of *S. marginata* was 935 bp shorter than that of the others and was entirely located in the SSC region. Relative synonymous codon usage (RSCU) was computed for protein-coding genes. These genes revealed 26,770 and 26,596 codons in *S. banzragczii* and *S. marginata* plastomes, respectively, and they had similar patterns in both plastomes, with the highest RSCU values for the AGA (1.9) codon of arginine (Arg) and the highest coding rate for leucine (Leu; 5,216 codons), isoleucine (Ile; 4,138 codons), and phenylalanine (Phe; 3,603 codons) (Fig. 4). Codons with A or T at the third position had a strong codon bias. The two species studied exhibited similar patterns. All coding sequences (CDS) contained a standard ATG start codon. Among the three stop codons, TAA was the most common. The sequence identities of the seven *Swertia* species were analyzed using mVISTA, with the *S. banzragczii* plastome serving as a reference. As expected, the genic regions were more conserved than the intergenic regions, and genes in the IR regions were highly conserved, followed by those in the LSC and SSC regions (Fig. 5).

**Fig. 6:**
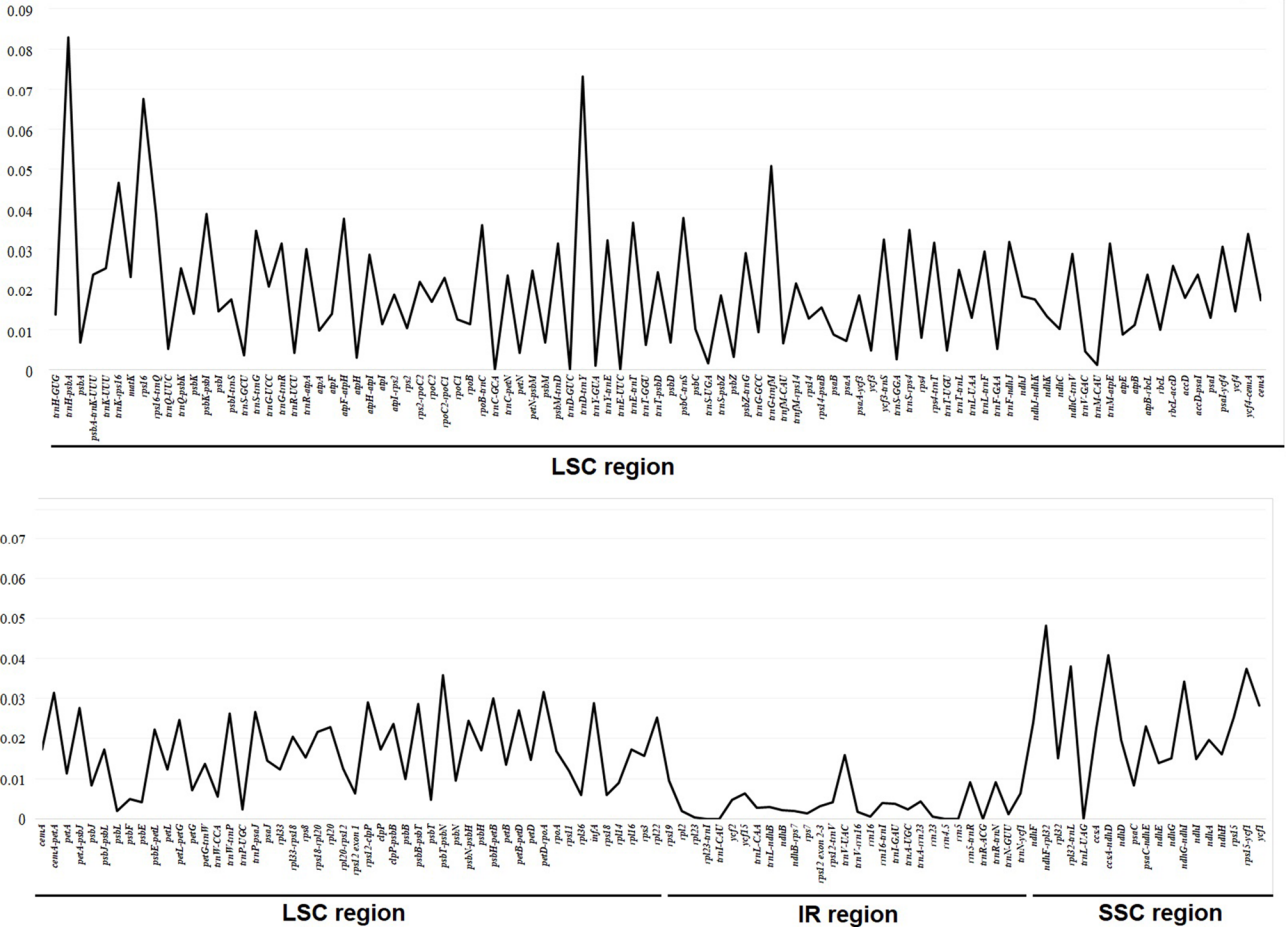
Comparison of the nucleotide diversity (Pi) values among the *Swertia* species. The mean Pi value is indicated by the blue line

We analyzed the genetic divergence of genes and intergenic regions in the *Swertia* genus. Overall, *Swertia* plastomes had an average genetic diversity (Pi) value of 0.17. The highest Pi values were observed in the *trn*H-UUU–*psb*A (0.082) and *trn*D-GUC–*trn*Y-GUA (0.016) intergenic regions of the LSC regions (Fig. 6). Among these genes, the highest value (0.67) was found for *rps*16 in the LSC region. The genic and intergenic regions in the IR region were relatively constant compared to those in the LSC and SSC regions. Common chloroplast barcoding markers such as *mat*K, *rbc*L, *trn*L-F, and *trn*H-*psb*A showed relatively low genetic diversity within the *Swertia* genus.

### Phylogenetic position of *S. banzragczii* and *S. marginata*

**Fig. 7:**
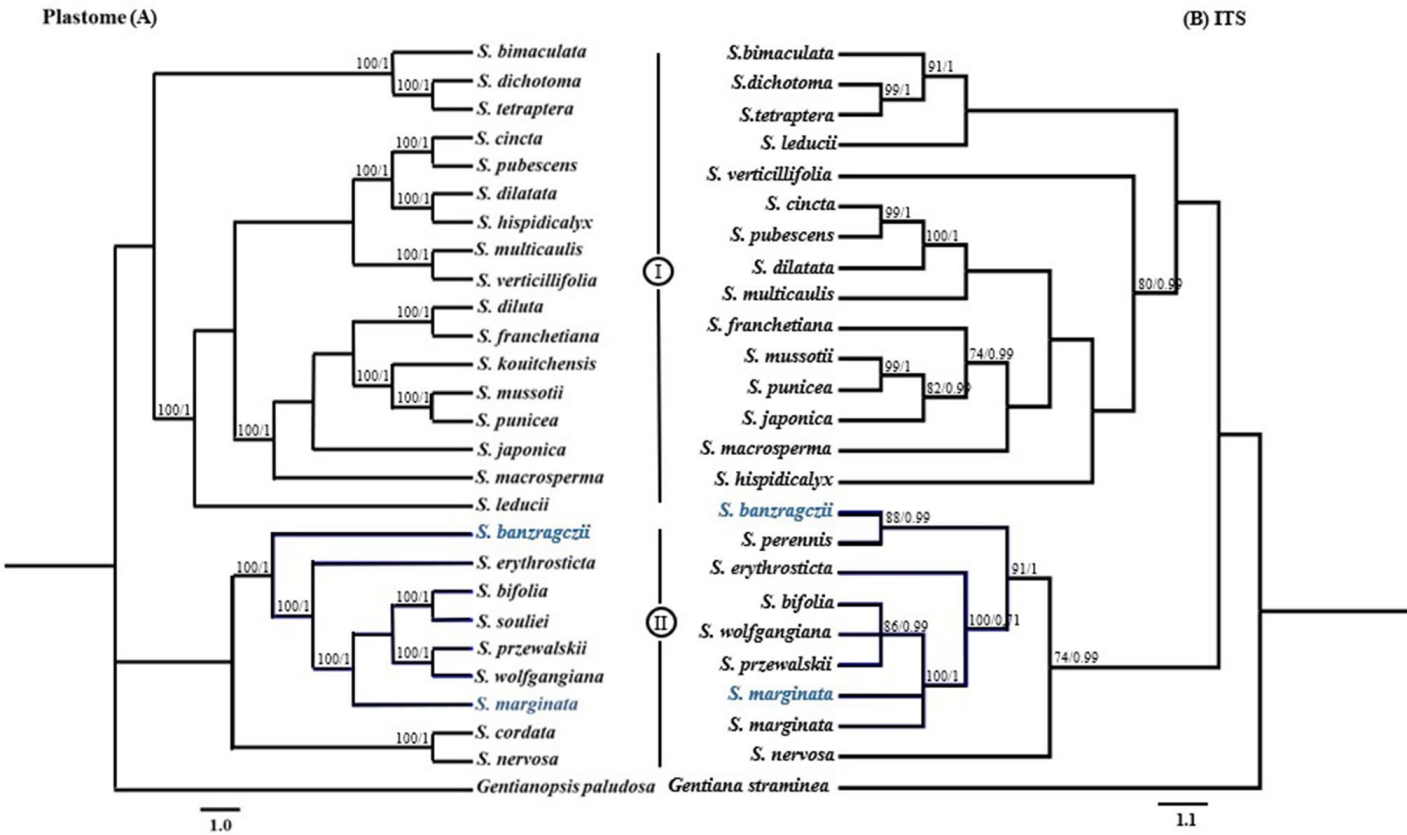
Phylogenetic tree of *Swertia banzragczii* and *Swertia marginata* with related taxa based on complete chloroplast genome (**A**) and nuclear ITS marker (**B**) Bayesian inference (BI) and maximum parsimony (MP) support values indicated at each branch, following the order BI/MP. The newly sequenced plastomes of each species are indicated in blue in both trees

The alignment of *Swertia* plastomes consisted of 152,075 bp, of which 82,024 bp (94.2%) were similar, and 5,051 bp (5.8%) were parsimony-informative. The alignment of the ITS sequences consisted of 622 bp, 153 of which were parsimony-informative. The topologies of maximum parsimony (MP) and Bayesian interference (BI) were highly congruent with the datasets and supported by strong bootstrap values and posterior probabilities. *Swertia* species were divided into two clades (I and II) based on the plastome and the ITS trees (Fig. 7). The clade I consist *S. banzragczii* and *S. marginata* with section *Swertia* including *S. bifolia*, *S. erythrosticta*, *S. souliei, S. przewalskii* and *S. wolfgangiana* (Fig. 7). *Swertia perennis* was used only in ITS tree and formed monophyletic group with *S. banzragczii*. The clade II include species from section *Swertopsis* and *Platynema*. However, the relationship of species within the subbranches produced incongruent topologies in the plastome and ITS tree.

## Discussion

The complete plastome of *Swertia* has recently been studied (Bi et al. 2020; Yang et al. 2020; Cao et al. 2022; Qing et al. 2022; Yang et al. 2022; Yang et al. 2023). However, previous studies have not explored Mongolian *Swertia* species. In this study, we sequenced and characterized the plastomes and determined their taxonomic positions based on the genetic and morphological features of two *Swertia* species in Mongolia.

The complete plastomes of *S. banzragczii* and *S. marginata* have a typical quadripartite structure with one LSC, one SSC, and two IR regions. They have relatively conserved features, genes, and GC content of sequences, similar to most species in Gentianaceae, and no significant structural rearrangements (Fig. 2) (Zhang et al. 2020; Yang et al. 2023). The *Swertia* plastome ranges from 149,089 bp (*S. cincta*) to 153,872 bp (*S. banzragczii*), and the difference in length is caused by expansion and contraction of the IR region, as previously reported in the *Swertia* genus (Cao et al. 2022; Yang et al. 2022). The IR lengths of *Swertia* ranged from 25,331 to 25,890 bp, and *rps*19 and *ycf*1 spanned the LSC/IRb and SSC/IRa junctions, respectively, within all species. The IRb/SSC junction varied greatly owing to the extension of 5–100 bp of *ndh*F from SSC to IRb. In the *S. banzragczii* plastome, the IRb/SSC junction includes *ndh*F, which consists of 54 and 2,175 bp in the IRb and SSC regions, respectively (Fig. 3). In contrast, the IRb/SSC junction of *S. marginata* plastome did not contain *ndh*F, which was located entirely in the SSC region. Synonymous codons are genetic codons encoding the same amino acids. Codon usage bias describes the frequency of synonymous codons that vary among different species. Codon usage patterns can be used to evaluate genetic evolution, environmental adaptation, and evolutionary relationships between species (Athey et al. 2017). Previous studies have shown that analysis of codon bias in the *Swertia* plastome revealed that leucine, isoleucine, and phenylalanine had the highest coding rates (Yang et al. 2022), and similar results have been reported in *S. banzragczii* and *S. marginata* plastomes (Fig. 4). In addition, the plastomes of the subtribe Swertiinae had a weak codon bias, which might be related to the conserved of plastome (Yang et al. 2023).

The plastome has a slower rate of nucleotide substitution in the coding region and a more conservative gene structure and content than the nuclear genome. It can act as a molecular marker for the interpretation of evolutionary relationships and improve phylogenetic resolution at lower taxonomic levels (Wicke et al. 2011). The sequence divergence of 23 *Swertia* species was discovered by Yang et al. (2022); five (*rpo*C1, *ccs*A, *ndh*I, *ndh*A, and *rps*15) protein-coding genes and five (*trn*H–*psb*A, *psa*A–*ycf*3, *cem*A–*pet*A, *ycf*15–*trn*L, and *ccs*A–*ndh*D) non-coding regions had the highest variability in the plastome. In addition, the plastomes of 26 *Swertia* species were used in this study to calculate genetic diversity; the *rps*16 gene and certain intergenic regions (*trn*H–*psb*A, *trn*D–*trn*Y-, *ndh*F–*rpl*32, *trn*K–*rps*16, *ccs*A and *ndh*D) showed the highest values (Fig. 5). Among these regiosn, *trn*H–*psb*A used to barcoding marker in previous phylogenetic studies of *Swertia* (Groff et al. 2015). Furthermore, Yang et al. (2022) reported that some species of *Swertia* have lost an exon of *rps*16, which has been found in several Gentianaceae species, and that its structure was similar in all species.

The phylogenetics of *Swertia* have been the subject of extensive research. Previous phylogenetic studies of *Swertia* species have used nrDNA (ITS) and plastid (*atp*B-*rbc*L, *mat*K, *trn*L, *trn*L-*trn*F, *trn*S-*ycf*9, and *trn*H-*psb*A) barcoding markers (Chassot et al. 2001; Xi et al. 2014; Groff et al. 2015). These findings indicate that *Swertia* is paraphyletic, with certain species sharing a foundational polytomy with other closely related genera. Moreover, pollen morphology analysis revealed that the *Swertia* genus has 14 different lineages at the respective phylogenetic positions within the Swertiinae subtribe (Chassot and Hagen 2008). Recently, the taxonomic relationships of *Swertia* have been revised using the complete chloroplast genome (Cao et al. 2022; Favre et al. 2010; Yang et al. 2022; Yang et al. 2023). Some studies have investigated the connections between *Swertia* and other genera in Gentianaceae, such as *Gentianopsis*, *Halenia*, *Lomatogonium*, *Lomatogoniopsis*, and *Sinoswertia* (Park et al. 2019; Li et al. 2021; Yang et al. 2023). These findings support the polyphyly of *Swertia*, which is consistent with previous molecular investigations but provides significantly better resolution. In this study, we first determined the phylogenetic position of *S. banzragczii* and *S. marginata*, which were formed the monophyletic clade with section *Swertia* species based on the plastome and nrDNA (ITS) phylogenetic trees (Fig. 7B). In previous studies, the section *Swertia* always formed a well-supported monophyletic clade, whereas the *Swertia* genus was polyphyletic in phylogenetic trees (Xi et al. 2014; Yang et al. 2022). The phylogenetic positions of the species within *Swertia* were inconsistent among the phylogenetic tree of plastomes and nrDNAs, specifically relationships of species within clade II. This incongruence was likely the result of different inherited backgrounds and mutation rates of ITS and plastome (Wolfe et al. 1987). More *Swertia* species need to be sequenced in further studies to better understand their phylogenetic relationships.

### Taxonomic notes on*S. banzragzcii* and *S. marginata*

In general, the morphological studies of both species were well recognized in original works (Sanchir 1984; Ho and Liu 2015). For example, morphological observations of *S. marginata* (including *S. komarovii*) were discussed based on detailed flower morphology and pollen grains (Ho and Liu 2015).

#### Swertia banzragczii

**Fig. 8:**
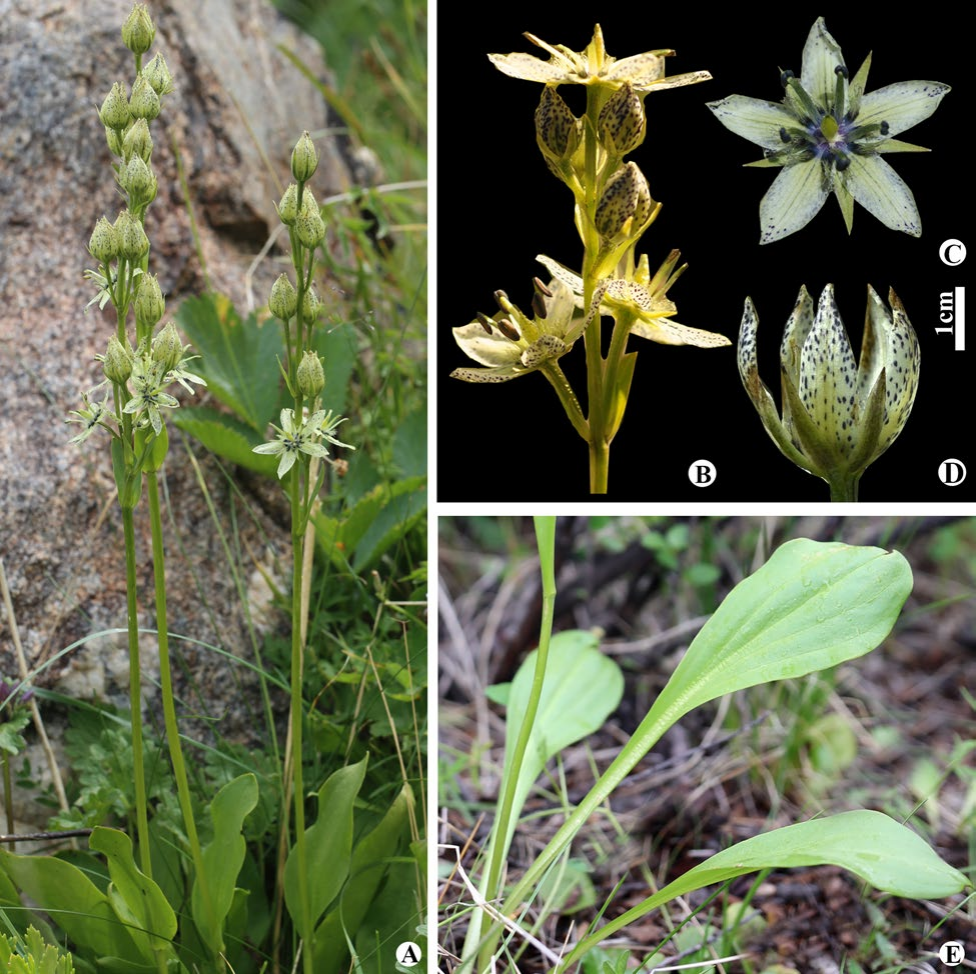
*Swertia banzragczii* in Mongolia: **A** general habitat; **B** inflorescence; **C** flower front view; **D** flower; **E** cauline leaves

Sanchir, Novosti Sist. Vyssh. Rast. 21: 136 (1984). (Fig. 8). TYPE: Bayan-Ulgii Province, Sagsai Soum, Dayan Lake, 26 July 1977, Ch. Sanchir, Z.V. Karamisheva, I.Yu. Sumerina, D., Baatarsukh 1273 (Holotype LE n.v.).

*Taxonomic notes. Swertia banzragczii* was first described in Western Mongolia (Sanchir 1984). Later, this species was found in Xinjiang, China (Chen, 2011); however, its taxonomic status of *S. banzragczii* is uncertain (Ho and Liu 2015). Based on molecular evidence, our results showed that *S. banzragczii* is a true species that its current Altai endemic species has a narrow distribution in the Altai Mountain range (Erst et al. 2022).

*Penology*. Flowering and fruiting in July to September.

*General distribution and habitat.* China (Xinjiang) and Mongolia (the Khovd and Mongolian Altai regions)(Baasanmunkh et al. 2022a). This species grows in alpine meadows, dwarf birch groves, sparse larch groves, and the borders of the alpine belt (Baasanmunkh et al. 2022b).

*Conservation status. Swertia banzragczii* was assessed as near-threatened at the global level, according to our previous study (Erst et al. 2023).

#### Swertia marginata

**Fig. 9:**
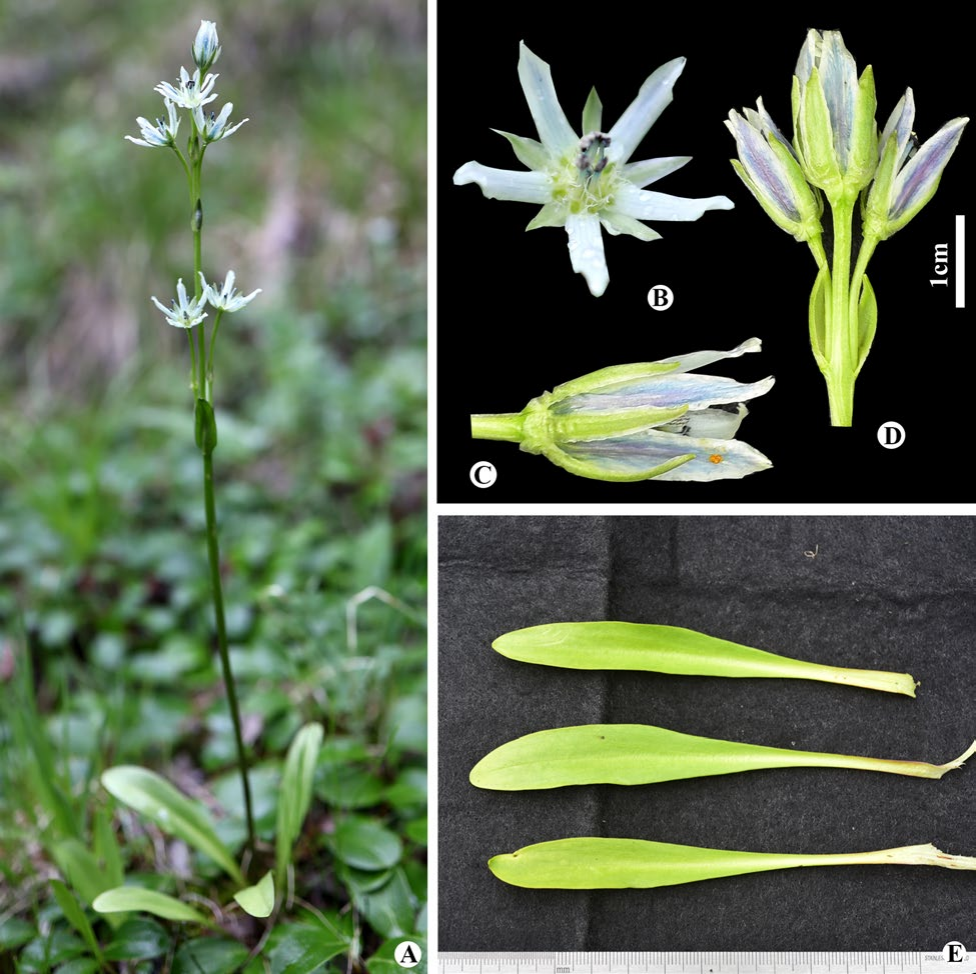
*Swertia marginata* in Mongolia: **A** general habitat; **B** flowers front view; **C, D** calyx; **E** cauline leaves

Schrenk, Bull. Sc. Acad. Petersb. x. 353 (1842). (Fig. 9) *Synonym*. *Swerita komarovii* Pissajuk. TYPE: Montes Sajanenses Orientalis, prope trajectum Carganskyi, 29–31 July 1902, fl. *s.n. V.L. Komarov*. (Holotype: LE photo!, LE01057204; http://re.herbariumle.ru/01057204). Montes Sajanenses Orientalis, prope trajectum Carganskyi, 29 July 1902, *s.n. V.L. Komarov*. (Isotype: LE photo!, LE01057205; http://rr.herbariumle.ru/01057205)

*Taxonomic notes*. The taxonomic status of *S. marginata* has been thoroughly addressed by Ho and Liu (2015). According to Ho and Liu (2015), *S. komarovii* Pissjauk. was treated as a synonym of *S. marginata* based on its morphological characteristics, but it is currently accepted as a species in GBIF (https://www.gbif.org/species/5596000). *Swertia komarovii* was first described in Russia and may have occurred in the Khusgul region of Mongolia (Pissjaukova 1961). Based on our ITS phylogenetic results (Fig. 7B), the Mongolian samples clustered with *S. marginata*; therefore, we confirmed that *S. komarovii* is a synonym of *S. marginata*.

*Penology*. Flowering and fruiting in July to September.

*General distribution and habitat*. Afghanistan, China, Kazakhstan, Kyrgyzstan, Mongolia (Khuvsgul and Mongolian Altai regions), Russia, and Tajikistan (Ho and Liu 2015). In Mongolia, this species grows in wet meadows, syrts (elevated watersheds), along the banks of brooks, moraines, shrubs, rubbly sites, slopes, taluses, and rocks in spruce forests.

*Conservation status.* The conservation status is currently not assessed at the global level.

## Conclusion

In this study, the plastomes of *S. banzragczii* and *S. marginata* from Mongolia were sequenced and assembled for the first time. The genome size, organization, and gene content were similar to those of other *Swertia* species. However, they varied slightly with respect to genome length and sequence of intergenic regions. Based on the phylogenetic analyses, they belonged to the subgenus *Swertia*. In general, our results demonstrated that *S. banzragczii* and *S. marginata* are true species and contribute more information towards resolving taxonomic ambiguities around *Swertia* species in Mongolia. Furthermore, this study used molecular evolution and phylogenetic analyses to determine the genetic connections within *Swertia*.

## Materials and methods

### Plant material, DNA extraction, and sequencing

Fresh leaves of *S. banzragczii* and *S. marginata* were collected from Bayan-Ulgii and Khuvsgul provinces, Mongolia, and voucher specimens (UBU0005243 and UBU0030443) were deposited in the herbarium of the National University of Mongolia, Mongolia. Photographs of each species taken in the wild were obtained from field surveys conducted by the authors. Total genomic DNA was extracted from the silica gel-dried leaf material using the CTAB method (Doyle and Doyle 1987). DNA purity and concentration were quantified using agarose gel electrophoresis and a NanoDrop 2000 spectrophotometer, respectively.

The nuclear ITS region (White et al. 1990) was used for amplification and sequencing. The PCR cycling parameters were 5 min at 94 °C, followed by 30 cycles of 45 s at 94 °C, 45 s at 56 °C, and a final 7 min at 72 °C. The PCR reaction (20 µL) includes 10X PCR buffer, 1.5 U Taq DNA polymerase, 0.2 mM dNTPs, 1 µM of each primer, and 100 ng genomic DNA. PCR products were subjected to gel electrophoresis and cleaned using a PCR clean-up kit. The purified PCR products were directly sequenced in two directions using the corresponding primers. Sequences were aligned using ClustalW (Thompson et al. 2003), and default settings and manual adjustments were made using BioEdit (Hall et al. 2011). Ambiguous nucleotide bases were corrected using the corresponding bases of sequences obtained using the reverse primer. The DNA sequences generated in this study have been deposited in GenBank (https://www.ncbi.nlm.nih.gov/).

The complete cp. genome library was constructed using the TrueSeq DNA Nano Kit protocol on the NectSeq 500 platform (Illumina, San Diego, CA, USA), following the extraction of total DNA. Trimmomatic software version 0.36 (Bolger et al. 2014) was used to eliminate adapter sequences and low-quality reads to mitigate biases in the analysis. A FastQC v. 0.11 (Antil et al. 2023) was used to assess the overall data quality by generating a base quality plot. The plot illustrates the distribution of the quality values across each cycle.

### Genome assembly and annotation

*De novo*assembly was conducted using various k-mers with NOVOplasty (Nicolas et al. 2017), using the default settings. A BLAST analysis was conducted following the assembly of the complete plastome or draft plastome to determine the species with which each contig (or scaffold) exhibited similarity. *S. banzragczii* and *S. marginata* plastomes were annotated using the online software GeSeqAnnotation of Organellar Genomes (Tillich et al. 2017). After initial annotation, the tRNA genes were further checked using tRNAscan-SE v1.21 (Lowe and Chan 2016). We manually calibrated the start and stop codons of coding sequences in the software Geneious Prime 2023.2.1 (https://www.geneious.com). The gene map was drawn using OrganellarGenomeDRAW (OGDRAW) (Lohse et al. 2007). The newly sequenced plastomes were deposited in GenBank under accession numbers OR643855 (*S. banzragczii*) and OR786442 (*S. marginata*).

### Comparative analysis

Gene rearrangements of *S. banzragczii* and *S. marginata* plastomes were detected based on collinear blocks using Mauve v2.4.0 (Darling et al. 2010). The SC/IR boundary shifts at the four junctions (LSC/IRa, IRa/SSC, SSC/IRb, and IRb/LSC) were compared using IRScope (https://irscope.shinyapps.io/irapp/) (Amiryousefi et al. 2018). The RSCU of the plastomes was analyzed using MEGA11 software (Tamura et al. 2021). RSCU < 1 indicated a codon used less frequently than expected, whereas RSCU > 1 indicated a codon used more frequently than expected. Genome divergence within the five *Swertia* species was identified using mVISTA (Frazer et al. 2004) in Shuffle-LAGAN mode. The plastome sequences were aligned using MAFFT ver. 7.388 (Katoh 2002). The protein-coding genes (coding regions), intergenic spacers, and introns (non-coding regions) of the *Swertia* plastomes were separately extracted to screen for polymorphic hotspots. Sliding window analysis was used to calculate the nucleotide variability (Pi) using DnaSP v.6.11 (Rozas et al. 2017). All the compared plastomes were downloaded from the GenBank database (Table 3). The step size was 200 bp with a 600 bp window length.

**Table 3:** The accession numbers and the sequence lengths of the specimens

	Species	Plastome		ITS		
		Sequence ID	Length (bp)	Sequence ID	Length (bp)	
1	*Swertia banzragczii*	OR643855	153,872	OR034100	740	
2	*S. marginata*	OR786442	152,968	OR019670	746	
3	*S. bifolia*	MZ261897	153,293	KC861298	634	
4	*S. bimaculata*	MW344296	153,751	LM644018	697	
5	*S. cincta*	MZ261898	149,089	MN561222	687	
6	*S. cordata*	NC_054359	153,429	-	-	
7	*S. dichotoma*	MZ261899	152,977	DQ317488	630	
8	*S. dilatata*	MW344298	150,057	JX569819	786	
9	*S. diluta*	NC_057681	153,691	-	-	
10	*S. erythrosticta*	MW344299	153,039	KF563976	625	
11	*S. franchetiana*	NC_056357	153,428	KC935872	625	
12	*S. hispidicalyx*	NC_044474	149,488	KP419964	627	
13	*S. japonica*	LC744566	153,208	LC645204	812	
14	*S. kouitchensis*	MZ261902	153,475	-	-	
15	*S. leducii*	NC_045301	153,015	MN561242	708	
16	*S. macrosperma*	MZ261903	152,737	MN561226	714	
17	*S. multicaulis*	NC_050660	152,190	FJ010799	724	
18	*S. mussotii*	KU641021	153,499	MN561214	695	
19	*S. nervosa*	NC_057596	153,690	FJ010794	757	
20	*S. przewalskii*	MW344305	153,160	AF255913	623	
21	*S. pubescens*	MZ261905	149,036	KC861341	625	
22	*S. punicea*	MZ261896	153,448	MN561219	688	
23	*S. souliei*	ON164641	152,804	-	-	
24	*S. tetraptera*	MW044653	152,787	JF978834	674	
25	*S. verticillifolia*	MF795137	151,682	KX549987	624	
26	*S. wolfgangiana*	MZ261906	153,090	AF255914	622	
27	*S. perennis*	-	-	AJ580550	824	
28	*S. marginata*	-	-	KC861304	624	
29	*Gentianopsis paludosa*	NC_050656	151,308	**-**		
30	*Gentiana straminea*	-	-	MF579735	627	

### Phylogenetic analysis

The nrDNA (ITS) and plastome sequences were used to determine the phylogenetic positions of *S. banzragczii* and *S. marginata* within the *Swertia* genus. We constructed plastome datasets for 26 *Swertia* species using *Gentianopsis paludosa* (Hook.f.) Ma as an outgroup. The ITS dataset was constructed using 25 ITS sequences and *Gentiana straminea* Maxim. as an outgroup. Additional sequences of the compared species were downloaded from GenBank, and the accession numbers are listed in Table 3. Plastome alignment datasets were filtered to remove ambiguously aligned regions using GBlock ver. 0.91.1 (Talavera and Castresana 2007). The best-fitting model for nucleotide substitutions was determined using the Akaike information criterion in jModelTest v2.1.10 (Darriba et al. 2012). Phylogenetic trees were constructed using the MP and BI methods. The MP analysis was performed using PAUP*v4.3.99.169.0 (Swofford and Documentation 1989). The branch-swapping algorithm for MP analysis was performed using tree bisection reconnection. The robustness of the tree was evaluated using 1000 bootstrap replication indices, and the consistency, retention, and composite indices were calculated. BI analysis was carried out using the MrBayes 3.2.6 (Ronquist et al. 2012) with two independent runs of four simultaneous chains executed for 5,000,000 generations using the Markov chain Monte Carlo algorithm. Trees were sampled every 5000 generations, and the first 25% were discarded as burn-in. The reconstructed trees were visualized using FigTree v.1.4.4 (Rambaut 2012).

## Data Availability

Not applicable.

## Abbreviations

LSC: Large single-copy
SSC: Small single-copy
IR: Inverted repeat
GC: Guanine-Cytosine
CDS: Coding sequences
tRNA: transfer RNA
rRNA: ribosomal RNA
RSCU: Relative synonymous codon usage
MP: Maximum parsimony
BI: Bayesian interference
